# Ocean acidification and global warming impair shark hunting behaviour and growth

**DOI:** 10.1038/srep16293

**Published:** 2015-11-12

**Authors:** Jennifer C. A. Pistevos, Ivan Nagelkerken, Tullio Rossi, Maxime Olmos, Sean D. Connell

**Affiliations:** 1 Southern Seas Ecology Laboratories, School of Biological Sciences and The Environment Institute, The University of Adelaide, South Australia 5005, Australia; 2 ENSAIA, 2 Avenue de la Forêt de Haye TSA 40602 54518 Vandoeuvre-les-Nancy, France

## Abstract

Alterations in predation pressure can have large effects on trophically-structured systems. Modification of predator behaviour via ocean warming has been assessed by laboratory experimentation and metabolic theory. However, the influence of ocean acidification with ocean warming remains largely unexplored for mesopredators, including experimental assessments that incorporate key components of the assemblages in which animals naturally live. We employ a combination of long-term laboratory and mesocosm experiments containing natural prey and habitat to assess how warming and acidification affect the development, growth, and hunting behaviour in sharks. Although embryonic development was faster due to temperature, elevated temperature and CO_2_ had detrimental effects on sharks by not only increasing energetic demands, but also by decreasing metabolic efficiency and reducing their ability to locate food through olfaction. The combination of these effects led to considerable reductions in growth rates of sharks held in natural mesocosms with elevated CO_2_, either alone or in combination with higher temperature. Our results suggest a more complex reality for predators, where ocean acidification reduces their ability to effectively hunt and exert strong top-down control over food webs.

Apex and mesopredators shape ecosystem structure and function through their control of prey populations^1^,^2^,^3^. Their influence on ecological communities is driven by direct (i.e. by predation) as well as indirect effects (e.g. the presence of a predator that leads to an alteration in prey behaviour interactions^4^). Predators often have cascading effects on ecosystems. A well-documented example is that of killer whale predation on sea otters and the consequences for kelp forests^5^, where killer whales mediate otter numbers whose predation on herbivorous sea urchins mediates the presence of kelp forests. The body size, metabolism and mobility of predators are strong determinants of the strength of such trophic cascades^6^. Alterations in the body size, metabolic demands, hunting tactics, density, and distribution of predators can therefore lead to changes that cascade through entire ecosystems^2^,^3^,^7^. Because of this important function, there has been a long-standing interest in understanding the impact of predators in both terrestrial and marine ecosystems^8^,^9^,^10^. However, we have entered an era where rapid environmental changes are affecting the functioning and persistence of many species. Changing climatic conditions are likely to lead to altered community compositions, population dynamics and ecosystem functioning^11^. The mechanisms by which apex and mesopredators are vulnerable to global change and the consequences for the ecosystems in which they live is a relatively new area of enquiry. While models have suggested decreases in body size and collapse of their populations^12^, there is a tremendous gap in empirical studies that have studied the underlying mechanisms and have tested how such predators may respond to multiple global stressors (but see^13^,^14^,^15^).

Global average sea surface temperatures are predicted to rapidly rise due to the greenhouse effect by 1°–3 °C in 2100 and this is in addition to an increase of ~0.76 °C in the last 150 years^16^. Increased temperature can have both negative and positive effects on a multitude of biological responses, including vertical and latitudinal range shifts, species interactions, and feeding, growth, survival and development rates^17^,^18^,^19^. However, warming will not occur in isolation, but in combination with ocean acidification which is predicted to decrease ocean pH by 0.3–04 units by the end of the century^16^,^20^. Most studies have focused on the effects of ocean acidification and climate change on marine invertebrates, with the few studies on fish largely restricted to small-bodied species^21^,^22^. Furthermore, many studies evaluate the effects of increased CO_2_ and temperature in isolation rather than in combination with factors that have a strong probability of altering the outcome of single factor effects. Indeed, studies on the interactive effects of warming and ocean acidification on the performance of larger predators such as sharks are very limited^14^, preventing us from better understanding their fate due to future change and how this might affect a change in the intensity of predation. Another concern is that studies regularly use unrealistic elevations of temperature or CO_2_ and that most studies are done over short time periods and under simple laboratory conditions requiring cautious interpretation when applied to natural conditions.

Several studies have shown effects of elevated temperature on fish metabolism and growth, with tropical species suggested to be more sensitive than temperate species (as tropical species have evolved in a more stable environment) due their narrower thermal reaction norm and as such reducing their ability to cope with temperatures above their thermal optimum^23^. While elevated temperatures enhance basal metabolic rates they can also raise respiratory demand leading to a reduced aerobic scope for activity such as feeding, digestion and predator avoidance and as such reducing available energy for growth and reproduction^24^.

Recent short-term studies (up to 2 months) on elasmobranch species have discovered a range of changes to shark physiology and behaviour as a function of elevated CO_2_. The epaulette shark (*Hemiscyllium ocellatum*), a species that exhibits exceptionally high tolerance to severe hypoxia, showed no effects of elevated CO_2_ on their metabolic performance^13^, although metabolic rates of embryonic bamboo shark (*Chiloscyllium punctatum*) were negatively affected^14^. A study on small-spotted cat sharks (*Scyliorhinus canicula*) indicated no changes in growth; however, alterations in blood chemistry and a shift in swimming patterns and increased lateralization were detected, suggesting some effects on elasmobranch physiology leading to altered behaviour^13^. Elevated CO_2_ also reduced survival in early juvenile bamboo sharks^14^, and reduced odour tracking behaviour in smooth dogfish (*Mustelus canis*) by avoiding food odour cues as well as displaying reduced attack behaviour^25^. However, long-term studies that provide an understanding of the interactive effects of elevated CO_2_ and temperature on the behaviour and physiology of large, long-lived predators such as sharks are clearly lacking.

Many predators rely on a variety of cues such as odour to locate their prey as part of their hunting and foraging strategy. This is especially important in nocturnal feeders that almost solely rely on this function^25^,^26^,^27^. Olfaction plays an important role in many predators’ ability to locate prey at a distance as odour cues disperse further than most other cues and it is often the first cue of many encountered^25^. Olfaction is also important for avoiding predators and chemosensory communication with conspecifics^28^. Recent studies have shown negative impacts of CO_2_ on olfaction in several fish species^21^,^29^,^30^,^31^. Since olfaction is an essential mechanism of the foraging strategy of many species, any disruption to this mechanism due to increased CO_2_ could leave animals vulnerable to malnutrition and predation and ultimately reduced growth and survival.

Here we test the potential effects of near-future ocean warming and acidification on a temperate shark species, the Port Jackson shark (*Heterodontus portusjacksoni*). This study aims to determine: (i) the extent to which temperature and/or ocean acidification modify somatic growth through altered foraging rates, when food supply is unlimited, (ii) the effects of CO_2_ on hunting behaviour through olfaction, and (iii) the interactive effects of elevated temperature and CO_2_ on shark growth in mesocosms containing natural habitats and prey, where sharks need to hunt for their food. The direct effects of ocean warming on physiological performance was assessed in the laboratory, while in large mesocosms we studied the longer-term effects on shark performance by integrating metabolic effects and potential CO_2_ effects on hunting behaviour under more natural conditions.

## Results

**Figure 1:**
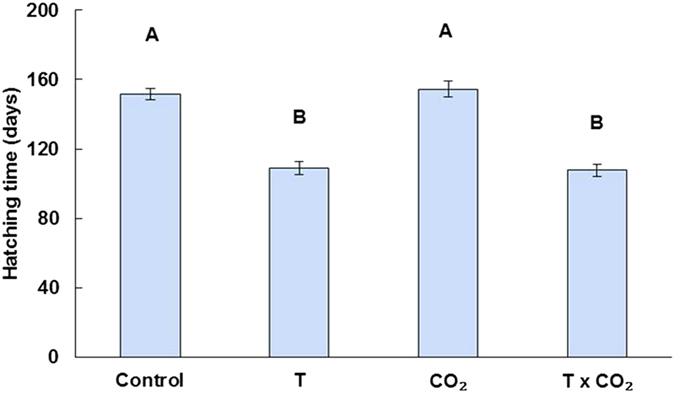
Mean duration until hatching for sharks eggs incubated in a factorial experiment of increased temperature (T) and CO_2_. Error bars represent ± 1 standard error of the mean. Bars with different letters (**A,B**) differ significantly (*P* < 0.05).

Elevated temperature increased the rate of embryonic development of sharks (Fig. 1, ANOVA; temperature, *F*_1,12_ = 49.565; *P* = 0.0001) but CO_2_ had neither an independent nor an interactive effect on hatch rates (Table S1) and 100% of the eggs hatched successfully with no mortality across any treatments. The forecasted end-of-century increase in temperature reduced the embryonic period by approximately 40 days out of 10–12 months on average. No significant differences in hatching size or weight were detected between treatments (*P* > 0.2).

**Figure 2:**
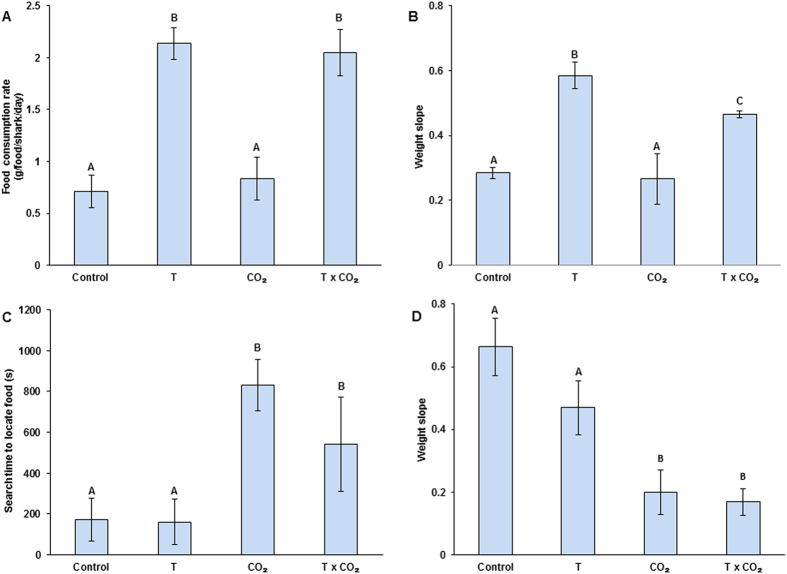
Shark food consumption rates, somatic growth rates, and hunting behaviour tested in a factorial design of elevated temperature (T) and CO_2_ as predicted for the end of this century. (**A**) Net food consumption rates in the laboratory where sharks were fed *ad libidum*. (**B**) Mean growth rates (slope of biomass increase over time) of sharks reared in the laboratory for 56 days on average and fed *ad libidum*. (**C**) Total time to successfully locate prey hidden in sand trays at the bottom of the mesocosms. (**D**) Mean growth rates (slope of biomass increase over time) for sharks reared in mesocosms with natural habitat and prey over a period of 68 days. For (**C**) and (**D**) the representative means are per tank/treatment. Error bars represent standard error of the mean, different letters represent significant differences (*P* < 0.05).

Newly hatched sharks were held under controlled laboratory conditions and fed *ad libidum* for 33–81days to determine the metabolic effects of temperature and CO_2_ on feeding and growth. Sharks tripled their food consumption rates under elevated temperatures compared to the control treatments, irrespective of normal or elevated CO_2_ (Fig. 2a, ANOVA; temperature, *F*_1,12_ = 49.566; *P* = 0.0001). The increased food intake in both temperature treatments resulted in significantly increased growth rates compared to the control (Fig. 2b, ANOVA; temperature, *F*_1,76_ = 62.733; *P* = 0.0001; temperature x CO_2_: *F*_1,76_ = 4.001; *P* = 0.0460). Whilst elevated temperature yielded the highest F-value for any term in the analysis accounting for most of the variation in the treatments, elevated CO_2_ had an antagonistic effect on growth when combined with the elevated temperature (Table S2). Nevertheless, growth under combined elevations of temperature and CO_2_ was still significantly higher than under control conditions (Fig. 2b).

To understand how hunting behaviour may be affected by the treatments, experiments were repeated in mesocosms mimicking a natural mini-ecosystem in which sharks had to locate familiar, but hidden prey. We observed that sharks reared under elevated CO_2_ (66–68 days in mesocosms) took almost 4 times longer than those in controls to locate their prey (Fig. 2c). However, in combination with an elevated temperature the time it took to locate prey was reduced by a third (although with a larger variance), which was still significantly higher than that for the control and elevated temperature only treatment groups (Table S3). All sharks in the control mesocosms approached the sand trays with hidden prey as soon as they were placed into the mesocosms and started shifting through the sand to find the food. However, under elevated CO_2_ and its combination with temperature not all sharks responded immediately, with 2 out of 9 sharks in the high CO_2_ treatment not responding to the introduction of prey at all. Additionally, for the sharks that responded there was a significantly higher failure rate (chi square test; χ^2^ = 27.88_219.9_) in terms of number of sharks that successfully located their prey in the elevated CO_2_ treatment and the elevated CO_2_ and temperature treatments (50% failure across sharks from both elevated CO_2_ treatments vs. 27% failure across both non-elevated CO_2_ treatments).

The reduced effectiveness of sharks to locate their prey through olfaction due to increased CO_2_ was reflected in their growth. Sharks reared for over 2 months in mesocosms with either elevated CO_2_ or elevated temperature and CO_2_ showed significantly lower growth rates (Fig. 2d, Table S3, ANOVA; CO_2_: *F*_1,29_ = 25.33; *P* = 0.0002) compared to the other treatments at ambient CO_2_ levels, where their growth was reduced by 70% in the elevated CO_2_ treatment and by 75% in the combined elevated temperature and CO_2_ treatment (Fig. 2d).

## Discussion

Our results show that ocean acidification and ocean warming can strongly govern embryonic duration, hunting behaviour, food consumption rates, and growth of a mesopredator. Impairment of effective foraging and growth may reduce the resilience and sustainability of predator populations. Under temperature forecasted for the end of the century, sharks increased their food consumption when fed *ad libidum*. However, when combined with the concurrent predicted elevation in levels of ocean CO_2_ there was a failure to allocate these resources towards maximal somatic growth. This indicates the presence of an antagonistic effect of CO_2_ on temperature reflecting a direct metabolic cost of increased CO_2_ in conjuncture with higher temperatures. With temperature-driven increases in metabolism, the likelihood of predator starvation increases when it is not matched by elevated ingestion rates; in some cases (such as the juvenile hammerhead) sharks are at provisioning limits and these stresses could push them into starvation^32^. A mismatch between food demands and food availability has for example been shown in low-productivity ecosystems^17^,^18^ and low-fertility systems^33^. Possible pathways of negative CO_2_ effects on animal physiology are a reduction in protein synthesis, and the costs of acid-base regulation or cardiorespiratory control^34^. Predator-prey relationships across marine ecosystems are strongly dependent on the body mass of the predator and prey and size-based predation is responsible for the transfer of energy across the food chain^35^. With increasing temperature, different sensitivities of species to rising CO_2_ might therefore lead to alterations in the body sizes of some predator species, which may have cascading effects on other species through altered predator-prey relationships.

Embryonic development time in Port Jackson sharks was reduced by temperature, but unaffected by elevated CO_2_, and with 100% survival in all cases. Faster development would result in reduced exposure times to egg predation which would increase their early life stage survival; like many fishes, sharks optimise energetics to favour early growth to reduce neonate and juvenile vulnerability. Port Jackson sharks usually suffer from very high embryonic mortality (89%) with 98% of the loss due to predation^36^. In contrast, elevated temperature and CO_2_ reduced hatching success in a temperate skate species (*Leucoraja erinacea*)^15^ and elevated temperature reduced juvenile condition and survival in a tropical shark (*C.punctatum)* as well as reduced embryonic survival (with no effect of pH on embryonic survival)^14^.

Organisms typically have some capacity to acclimate to potential stressors either by altering aspects of their physiological, behavioural or morphological characteristics to enable them to cope with changes^37^. Some are more permanent alterations (developmental acclimation) whereas others are reversible. Many studies use juveniles or adults and expose them to high temperature and or elevated CO_2_ for a short period of time and cannot realistically account for within-generation acclimation, including developmental acclimation^37^. We provide the first insight into within-generational acclimation potential by exposing sharks from their embryo stage through to their juvenile stage to two major global stressors both in a laboratory and in a mesocosm setting. Importantly, after more than seven months of experimental exposure we find no clear signs of acclimation over this critical period of growth and survival. Recent studies have shown only partial acclimation to elevated temperature and CO_2_ for metabolic rates and growth in fish when parents experience the same stressors as the offspring^37^,^38^, but this was not the cause for behaviour^39^. It is therefore highly unlikely that our sharks, which are slow growing, long-lived animals, would show any significant acclimation at a later developmental stage. It is important to note that the temperature within our mesocosms was 1 °C higher and the *p*CO_2_ was approximately 300 ppm lower than in our laboratory experiment and this is important because negative behavioural effects were still detected at these levels (~700 ppm) which will be reached before the end of the century based on the current CO_2_ emission trajectory^16^. Moreover, shallow coastal habitats that naturally experience naturally high CO_2_ levels from upwelling and/or eutrophication^40^ will reach predicted levels sooner than open oceans^41^.

Impacts of global change stressors could alter survival (through altered anti-predator behaviour) as well as foraging success in mesopredators and thus directly affect upper and lower trophic levels^42^,^43^. Detecting sufficient prey in a large aquatic environment is difficult and sharks and other aquatic predators have evolved a variety of senses to aid prey detection^26^, with odour taking a primary role in the sensory hierarchy^25^. This is especially true for predators that hunt at night to avoid predation pressure. Odour is an important cue in aquatic environments as it can disperse further and be detected sooner than any other cue, especially as a directional cue (vision: < 100 m, sound: 25–150 m, odour: up to 10 km^44^). Here we show that sharks exposed to elevated CO_2_ levels were slower and less successful in finding prey through olfaction (as prey was dead and thus electroreception can be excluded) and that this resulted in significantly reduced growth in mesocosms that mimicked natural environments. Failure in detection of olfactory cues due to elevated CO_2_ has been observed in smooth dogfish (*M. canis)*, but this study did not include tests of the effect of ocean warming^29^. Sensory failure due to ocean acidification could affect predators in several ways. Reduced olfactory capacity would leave some prey items undetected, while predators might spend more time actively searching to compensate for reduced prey capture success. It would also make them vulnerable to higher-order predators, for example towards ambush predators such as the wobbegong, pinnipeds, and even other fish during their juvenile phase^45^. If mesopredators altered their nocturnal hunting to daytime hunting strategies to rely more on visual cues than just olfactory cues they would be more susceptible to predation as well. Elevated CO_2_ has been shown to alter the nocturnal swimming pattern of small-spotted cat sharks as well as significantly increase lateralization^13^ providing further support to potentially altered hunting strategies. At the same time predators need to cope with the increased energetic demands due to elevated temperature, as well as with the increased metabolic costs of CO_2_. Predators may adapt to olfactory disruption by relying more on other senses to detect prey (e.g. vision, electroreception, mechanoreception), but these may be affected by ocean acidification as well^46^ and because these typically detect cues at shorter distances, search times may increase and successful prey capture may decrease leading to lower food intake with consequences for their fitness. Reduced predator detection and recognition by mesopredators likely increases mortality, and any alteration of their anti-predator behaviour comes at a cost of other behaviours such as foraging.

Future ocean warming and acidification will not be uniform across the globe due to the interaction of multiple climatic and non-climatic factors at local spatio-temporal scales^47^, and it is at these scales that organisms are most affected. In regions where temperatures rise at relatively higher rates than CO_2_, predators such as sharks may grow faster due to higher food intake rates, but the outcome will be highly dependent on food availability. With the predicted reductions in abundances of many species at intermediate and lower trophic levels^48^, the energetic demands of large predators may not be met. In areas with relatively more rapid increases of CO_2_ rather than temperature, predators might not meet their energetic requirements either, but through alternative mechanisms, i.e. reduced effectiveness in locating prey. Additionally, the potential for sharks to migrate would also influence the type of ecological impacts these stressors impose as sharks are highly mobile species that are able to move vast distances^49^. Sharks may be able to relocate to a more suitable habitat or higher latitudes thus affecting the strength of their interaction within the systems they leave behind and introduce new pressures to the new habitats they occupy^42^. Our parallel laboratory and mesocosm approach is not able to evaluate such changes, but range shifts could mitigate the negative effects on some shark populations.

One third of shark and ray species are threatened worldwide^50^. Their life histories of late sexual maturation and slow reproduction rates followed with long gestation periods result in very low population growth rates making them highly sensitive to elevated fishing mortality^51^. While overfishing remains the greatest direct threat on shark populations, the additive effects of increasing ocean acidification and warming is likely to further exacerbate their demise^42^,^50^,^52^. Considering that both stressors will increase concurrently, the implications for populations of high-order carnivores are likely to be more considerable than estimates derived from single-factor studies on sharks. This has important management implications for their populations. Since it is not possible to reverse the effects of climate change and ocean acidification in the short term, the importance of reducing fishing mortality of large-bodied predators are even greater on the short-term.

**Figure 3:**
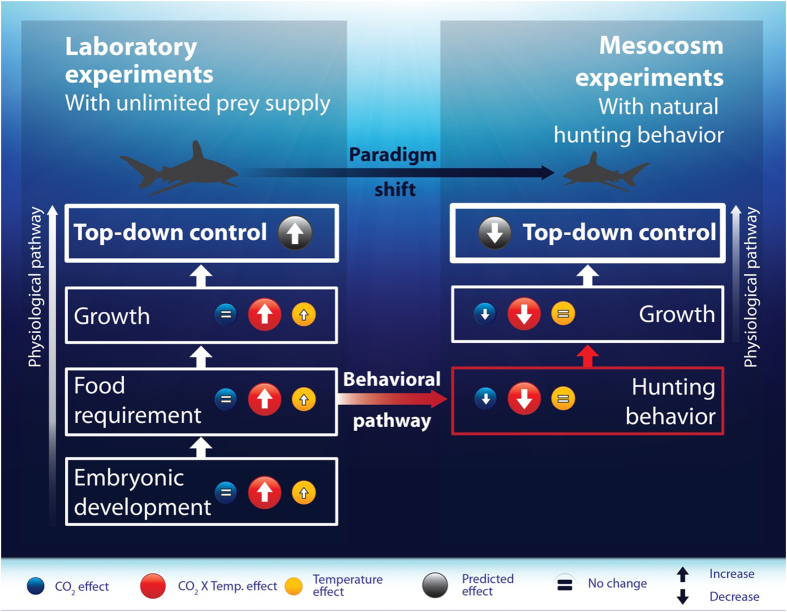
Conceptual diagram showing the individual and interactive effects of elevated temperature and CO_2_ on the physiology (development rate, food consumption rate, and growth rate) and behaviour (hunting for prey through olfaction) of sharks, based on the results of our long-term laboratory and mesocosm experiments. Arrows within circles show whether the respective factors increase, decrease, or remain the same. Left-hand panel shows results that support the current predicted increase in energetic demands by predators leading to a potential increase of top-down control on food-webs. Right-hand panel shows our suggested paradigm shift linked to a negative effect of CO_2_ on olfaction-driven predation. CO_2_ leads to a reduced effectiveness in finding prey, leading to reduced growth, and therefore negates the predicted increase in top-down control based on elevated temperature alone. Artwork by T. Rossi.

With elevated temperatures leading to higher metabolic rates and the need for higher food intake, predators may exert a stronger control on their prey populations due to climate change^10^. We challenge this model because CO_2_ may negate these temperature effects by reducing the effectiveness of hunters to successfully capture prey and exert such top-down control (Fig. 3). A reduction under future climate conditions in the growth rates of mesopredators, as demonstrated in our mesocosm experiment, could therefore potentially lead to modified predator-prey interactions^53^ and have cascading effects on food web structure^54^. Depending on the species and their role in the ecosystem, reduced predator influence could lead to weakened top-down control over prey allowing lower-order consumers to increase in abundances and affecting their prey species. This would be primarily true for predators that rely on olfaction as a sense to find prey, particularly sharks. Rather than an increase in top-down control as is currently predicted, our results suggest a more complex reality for predators, where ocean acidification reduces their ability to effectively hunt and exert strong top-down control over food webs.

## Materials and Methods

### Ethics statement

Research was carried out under approval of the University of Adelaide animal ethics committee (permit: S-2013-095) and according to the University’s animal ethics guidelines. Egg collections around the Gulf St. Vincent were carried out with permission of the South Australian Government Department of Primary Industry and Regions SA (permit: 9902595).

### Study species and sample collection

The study species *Heterodontus portusjacksoni* (Meyer, 1793) is an ideal model species because it is robust to handling stress that could affect their physiology^55^. It is a medium-sized benthic oviparous shark endemic throughout the southern half of Australia^56^. It is known to aggregate in groups as juveniles, however, this is also influenced by habitat^57^. It breeds annually, between the months of September and November, laying a pair of eggs every 10–12 days over 2–3 month period^27^ and the incubation can last up to a year. *H. portusjacksoni* lays large eggs containing a single embryo with an average weight of 155.5 g^56^. A total of 98 eggs were collected from Gulf St. Vincent, South Australia, over two collection dates (7th and 28th June 2013) via snorkelling.

### Egg and shark rearing

The collected eggs were held in a temperature-controlled laboratory until hatching. Developmental stages^56^ were determined for all collected eggs and showed that they were of similar stage (stage 14—at least 7.5 months). The eggs were placed in 40 L tanks containing natural filtered seawater which was partially exchanged every 2–3 days. The tanks were placed in water baths with temperatures maintained using heater chiller units (TR15 Aquarium chillers, TECO refrigeration technologies, Ravenna, Italy), and 300 W glass heaters. Pumps were connected to the chiller units which ensured an even temperature distribution throughout the water baths. The eggs were left to acclimatize over a period of seven days where temperature was steadily increased by 1 °C to the elevated temperature treatment. The eggs were kept in either control (~400 μatm) or elevated CO_2_ (~1000 μatm)^16^,^58^ crossed with control (~16 °C) or elevated temperature (~19 °C) (Table S4). Eggs were evenly distributed over 4 tanks per treatment with a max density of 9 eggs per tank. Exposure time of the embryos varied from an average of 108 days for the elevated temperature treatment to 143 days for the lower temperature treatments (hatching rate was affected by temperature which affected embryonic exposure time).

Upon hatching the juvenile sharks were relocated to new tanks with the exact same treatment set-up as described above, again with 4 tanks per treatment. The sharks were placed into large tubs of 100 L or 150 L in volume. The number of sharks in each of the tanks ranged from 1–8 for the 150 L tanks and 1–4 for the 100 L tanks (differing numbers due to differences in hatching time and because at some point 33 sharks were removed for the subsequent mesocosm experiment). Sharks were kept in their respective treatments for at least 2 months. Water parameters (Table S4) were measured daily. Tanks received water changes every other day (minimum 40% of total volume). Sharks were fed *ad libidum* with thawed frozen prawns daily.

A PEGAS 4000 MF Gas Mixer (Columbus Instruments, Columbus, Ohio) was used to achieve different CO_2_ concentrations in the seawater by bubbling the CO_2_ enriched air directly into the tanks. The gas mixer was connected to a CO_2_ tank and an air compressor. Temperature and pH_NBS_ of each tank was measured daily using a pH and temperature meter (Mettler Toledo SevenGo™ SG2) calibrated with fresh buffers each day. Additionally, oxygen and salinity were also measured daily within the tanks. Total alkalinity of seawater was estimated by Gran titration (888 Titrando, Metrohm, Switzerland) from water samples taken weekly from each of the treatment tanks. Alkalinity standards were accurate within 1% of certified reference material from Dr A. Dickson (Scripps Institution of Oceanography; Langdon *et al*. 2000). Average seawater *p*CO_2_ (Table S4) was calculated using CO2SYS with the constants of Mehbrach *et al*.^59^ refit by Dickson and Milero^60^. The variability in *p*CO_2_ is higher than for pH because it was calculated using weekly measurements of total alkalinity, whereas pH was measured on a daily basis.

### Hatching rate, feeding and growth measurements in the laboratory

The tanks holding the eggs were checked daily for new hatchlings. As soon as new hatchlings were observed, their weight and sex was recorded as well as a photo taken of each individual for future identification. The newly hatched shark was then placed into a new tank with the same CO_2_ and temperature treatment as it experienced while still in the egg. The sharks were measured each week for changes in weight (±1 g) and also photographed to aid identification of individuals to track their growth for the duration of the experiment. Sharks were fed *ad libidum* with mussels and prawn meat during the first month after hatching, and afterwards with prawn meat alone. Food consumption was recorded daily by comparing the difference in weight of food offered and food remaining after 30 minutes of feeding. Thirty minutes was selected as the end period because this was well beyond the time it took sharks to feed to satiation (usually ~10 min). Because multiple sharks were kept in a tank, food consumption was calculated at the level of tanks and divided by the number of sharks in the respective tank. This was deemed as a fair representation of individual shark consumption rates because leftover food in the tanks indicated they were all fully fed and competition for food resources was unlikely to take place because food was not limiting. Although Port Jackson sharks usually feed at night, our sharks were conditioned to feed during the day directly upon hatching and therefore we expect this to represent true demand of food intake.

### Growth in mesocosm experiments

After the laboratory experiment, a subset of the sharks was relocated to a mesocosm setup in South Australia. Three sharks were placed in each of the 12 mesocosm tanks (2,000 L volume each) which were manipulated to mimic a shallow temperate reef habitat (n = 3 sharks for each control and treatment mesocosm, see Table S4). The mesocosms had the same crossed design of elevated CO_2_ and temperature as the laboratory experiments with 3 replicate mesocosm per treatment (Table S4). Each mesocosm had the same biological set up which included 5 kelp plants (*Ecklonia radiata*) with an average weight of 250 g per plant, a single spiny rock lobster (*Jasus edwardsii*) of ~2 kg in weight, 1 crab (*Ozius truncatus*), 15 snails (*Turbo undulatus)*, 6 urchins (*Heliocidarcis erythrogramma*) and amphipods (>1,000). The kelp, snails and urchins were replenished 3 times over the duration of the experiment (68 days) as needed. The snails, crab, lobster and urchins were too large for the sharks to consume, and their primary food source was the amphipods that successfully populated and reproduced within the tanks. Turf algae started growing naturally and covered the major part of the substratum in the mesocosms. The mesocosms had a flow-through system using natural seawater filtered through a sand filter. Temperatures were manipulated using external heater/chiller units (TC60 Aquarium chillers, TECO refrigeration technologies, Ravenna, Italy). The same thermal mass flow meter/controller as in the laboratory experiments was used to achieve an elevated CO_2_ concentration in the seawater of the mesocosm via bubbling of enriched air directly into the tanks, and both temperature and pH were measured daily.

The sharks were measured individually for total weight and photographed (to aid with the identification and tracking of individual growth for the duration of the experiment) prior to placement in the mesocosms. Sharks were re-measured after 61 days and after 68 days at the end of the experiment. During the first two weeks of the experiment, the sharks in both high temperature treatments were fed 2 g of fresh prawn meat, whereas sharks in both ambient temperature treatments were fed 1 g of meat each. These were similar to the food intake quantities as measured in the laboratory prior to placement into the mesocosms. This served as an acclimation period during which the sharks could familiarize themselves with the natural prey items in the mesocosms. After 2 weeks the feeding was standardized to 1 g per shark for all treatments. Due to the lowered food provisioning and due to their continuing increase in growth, the sharks increased their reliance on foraging on natural prey in the mesocosms such as amphipods. Observations showed shark foraging in-between the turf algae (which occupied most of the substratum and vertical tank walls of the mesocosms). Biomass of amphipods was not enhanced in the control treatments compared to the elevated CO_2_/temperature treatments (single sampling event of total weight and numbers of all amphipods found on the kelp: Control = 0.06 g, n = 124, Temperature = 0.06 g, *n* = 56, CO_2_ = 0.09 g, *n* = 86 and T × CO_2_ = 0.05 g, *n* =* *114) and could therefore not have been responsible for the observed reductions in growth rates in the latter treatments. There were no large differences in growth between the three sharks in one tank (this was the same for all tanks and no shark was observed to compete while feeding individually).

### Hunting behaviour in mesocosm experiments

After an average 36-day exposure (range: 35–38 days because sharks were introduced into the mesocosms over a 4 day interval) to the experimental treatments in the mesocosms, the effect of elevated CO_2_ on shark prey hunting behaviour through olfaction was tested. Prior to the day of testing the sharks were not fed, although they were still able to obtain prey (amphipods) from the tanks. Nevertheless, the sharks showed high degree of motivation towards the food offered in the olfactory trial the next day. The olfaction tests consisted of placing two equally sized (33 × 23 × 5 cm) sand-filled trays within each of the mesocosms. One tray (i.e. the food tray) had a combination of prawn meat (4 equally sized pieces of approx. 1 g each) and 5 fresh cockles of equal size (still in their shell but opened), buried into the sand. The control tray contained no food but had 5 empty and cleaned out cockle shells buried in the sand to reduce any visual bias of the slightly exposed top ends of the shell in the food tray. Both trays were placed near each other (average distance of 5 cm between the trays) and the shark responses were recorded using a GoPro HD HERO3 video camera (white edition) for a period of 40 min. The recordings were then analysed to determine the length of time it took for each shark to locate the hidden food and to determine the number of sharks that responded to the introduction of the prey. The timer started counting from the moment the trays were lowered onto the bottom of the mesocosm until the time each shark found the hidden prey items in the tray and started retrieving them from the sand or until the end of the experiment (after 40 minutes). Although Port Jackson sharks are nocturnal feeders these sharks were accustomed since birth to being fed during the day and responded actively when food was offered. It was possible to distinguish individual sharks within each mesocosm due to the markings on their upper bodies between the eyes, first dorsal and pectoral fins. These areas showed the most variation in patterning between sharks and remained consistent from hatching (photos were taken weekly after hatching).

### Statistical analysis

Separate linear regressions estimated individual growth of sharks over time. The slopes of each regression per shark was used for statistical analyses of factorial treatments; PERMANOVA version 1.0.3 (Anderson, 2005) that tested the effects of elevated CO_2_, temperature, and their interactive effects on growth, food consumption and hunting behaviour of the 2 × 2 factorial experiments. Subsequent pair-wise tests were used to determine the specific significances of each separate treatment combination. A significant tank effect was found for the hatching (Table S1) and consumption data (Table S2) only, this was not significant in any subsequent behaviour trials and on growth. For behaviour: tank did not have a significant effect when nested in factors temperature and CO_2_ and the statistical test was thus rerun without tank nested as a factor.

## Additional Information

**How to cite this article**: Pistevos, J. C. A. *et al*. Ocean acidification and global warming impair shark hunting behaviour and growth. *Sci. Rep*. **5**, 16293; doi: 10.1038/srep16293 (2015).

## Supplementary Material

Supplementary Information
