# Supplementary material for: Ocean acidification and global warming impair shark hunting behaviour and growth
Source: Sci Rep. 2015 Nov 12;5:16293. doi: 10.1038/srep16293 (PMC4642292; doi:10.1038/srep16293)
Supplement: Supplementary Information [file srep16293-s1.doc]

**Supplementary Information**

**Ocean acidification and global warming impair shark hunting behaviour and growth**

**Short title: Climate stressors impair hunting and growth in a large predator**

Jennifer C.A. Pistevos^1^, Ivan Nagelkerken^1*^, Tullio Rossi^1^, Maxime Olmos^2^, Sean D. Connell^1^

^1^*Southern Seas Ecology Laboratories, School of Biological Sciences and The Environment Institute, The University of Adelaide, South Australia 5005, Australia*

^2^ *ENSAIA, 2 Avenue de la Forêt de Haye TSA 40602 54518 Vandoeuvre-les-Nancy, France*

*Corresponding author: Ivan Nagelkerken, Southern Seas Ecology Laboratories, DX 650 418, School of Biological Sciences and The Environment Institute. The University of Adelaide, South Australia, SA 5005, Australia,

Phone: +61 8 8313 4137

email: ivan.nagelkerken@adelaide.edu.au

**Supplementary Information**

**Table S1:** Analyses of variance of the effects of temperature (target: 16 °C and 19 °C) and CO_2_ (target: 400 and 1000 ppm) on shark egg hatching rates.

| Source | DF | MS | F | | P | |
| --- | --- | --- | --- | --- | --- | --- |
|  |  |  |  | |  | |
| T | 1 | 30535 | 49.565 | | **0.0001** | |
| CO_2_ | 1 | 5.00 | 8.12 x 10^3^ | | 0.9330 | |
| T x CO_2_ | 1 | 14.364 | 2.33 x 10^2^ | | 0.8758 | |
| TK (T x CO_2_) | 12 | 8003.4 | 2.154 | | **0.0206** | |
| Residual | 75 | 23227 |  | |  | |
| Total | 90 |  |  | |  | |
|  |  |  |  |  | |  |

T = elevated temperature treatment, CO_2_ = elevated CO_2_ treatment, T x CO_2_ = combined elevated temperature and elevated CO_2_ treatment, TK = tank nested within T x CO2 interaction term. Degrees of freedom (DF), mean squares (MS), the *F*-ratio (F), *P*-value (P). Bold values indicate significance at p< 0.05.

**Table S2:** Analyses of variance of the effects of temperature (target: 16 °C and 19 °C) and CO_2_ (target: 400 and 1000 ppm) on (a) food consumption rates and (b) growth rates for sharks reared over 56 days, on average, in the laboratory.

| Source | DF | MS | F | | P | |
| --- | --- | --- | --- | --- | --- | --- |
| (a) Consumption |  |  |  | |  | |
| T | 1 | 318.25 | 49.566 | | **0.0001** | |
| CO_2_ | 1 | 3.80 x 10^-2^ | 5.92 x 10^3^ | | 0.9415 | |
| T x CO_2_ | 1 | 2.028 | 0.316 | | 0.5801 | |
| TK (T x CO_2_) | 12 | 6.563 | 9.568 | | **0.0001** | |
| Residual | 766 | 0.686 |  | |  | |
| Total | 781 |  |  | |  | |
| (b) Growth |  |  |  | |  | |
| T | 1 | 1.259 | 62.733 | | **0.0001** | |
| CO_2_ | 1 | 8.89 x 10^-2^ | 4.427 | | **0.0365** | |
| T x CO_2_ | 1 | 8.03 x 10^-2^ | 4.001 | | **0.0460** | |
| Residual | 76 | 2.01 x 10^-2^ |  | |  | |
| Total | 79 |  |  | |  | |
|  |  |  |  |  | |  |

T = elevated temperature treatment, CO_2_ = elevated CO_2_ treatment, T x CO_2_ = combined elevated temperature and elevated CO_2_ treatment, TK = tank nested within T x CO2 interaction term. Degrees of freedom (DF), mean squares (MS), the F-ratio (F), *P*-value (P). Bold values indicate significance at p< 0.05.

**Table S3:** Analyses of variance of the effects of temperature (target: 16 °C and 19 °C) and CO_2_ (target: 400 and 1000 ppm) for sharks reared in the mesocosms on (a) search time to locate prey, and (b) growth rates over 68 days.

| Source | DF | MS | F | P |
| --- | --- | --- | --- | --- |
| (a) Search time |  |  |  |  |
| T | 1 | 2.82 x 10^-5^ | 2.5195 | 0.1292 |
| CO_2_ | 1 | 8.44 x 10^-5^ | 7.5297 | **0.0120** |
| T x CO_2_ | 1 | 4.22 x 10^-5^ | 3.7654 | 0.0660 |
| Residual | 19 | 1.12 x 10^-5^ |  |  |
| Total | 22 |  |  |  |
| (b) Growth |  |  |  |  |
| T | 1 | 0.101 | 2.20 | 0.1446 |
| CO_2_ | 1 | 1.165 | 25.33 | **0.0002** |
| T x CO_2_ | 1 | 5.31 x 10^-2^ | 1.16 | 0.2982 |
| Residual | 29 | 4.60 x 10^-2^ |  |  |
| Total | 32 |  |  |  |

T = elevated temperature treatment, CO_2_ = elevated CO_2_ treatment, T x CO_2_ = combined elevated temperature and elevated CO_2_ treatment. Degrees of freedom (DF), mean squares (MS), the F-ratio (F), *P*-value (P). Bold values indicate significance at p< 0.05.

**Table S4:** Mean (± SE) seawater parameters in the experimental systems with two crossed factors of elevated temperature and CO₂ for the (a) shark eggs (b) sharks in the laboratory and (c) sharks in the mesocosms. Numbers in brackets following the treatment names represent the number of sharks in each treatment. N= no of replicates for pH readings, n = no of replicates for alkalinity readings The SE in S4 represents the variability of both replicates and measurements.

|  | Salinity | pH_NBS_ | Temp (ºC) | N | TA  (μmol.kg^-1^SW) | pCO_2_ (ppmv) | SE | n |
| --- | --- | --- | --- | --- | --- | --- | --- | --- |
| (a) Egg stage | |  |  |  |  |  |  |  |
| Control (24) | 40 (±0.0) | 8.02 (±0.01) | 16.3 (±0.0) | 143 | 2377.9 (±49.4) | 517.6 (±19.9) | 19.9 | 11 |
| T (20) | 40 (±0.0) | 8.06 (±0.00) | 19.2 (±0.1) | 98 | 2528.9 (±32.2) | 531.8 (±22.4) | 22.4 | 8 |
| CO₂ (23) | 40 (±0.0) | 7.82 (±0.01) | 16.2 (±0.1) | 136 | 2400.2 (±67.1) | 946.7 (±39.7) | 39.7 | 11 |
| T x CO₂ (27) | 40 (±0.0) | 7.81 (±0.01) | 19.1 (±0.1) | 118 | 2442.2 (±42.0) | 1048.5 (±45.9) | 45.9 | 10 |
| (b) Laboratory | |  |  |  |  |  |  |  |
| Control (24) | 40 (±0.0) | 7.96 (±0.01) | 16.4 (±0.1) | 73 | 2346.1 (±102.5) | 589.4 (±50.9) | 50.9 | 3 |
| T (20) | 40 (±0.0) | 7.87 (±0.02) | 18.8 (±0.3) | 55 | 2179.0 (±61.9) | 661.0 (±15.4) | 15.4 | 3 |
| CO₂ (25) | 40 (±0.0) | 7.69 (±0.01) | 15.9 (±0.1) | 73 | 2075.0 (±29.8) | 1003.6 (±69.9) | 69.9 | 3 |
| T x CO₂ (27) | 40 (±0.0) | 7.68 (±0.01) | 18.7 (±0.1) | 56 | 1944.4 (±98.1) | 1014.3 (±115.0) | 115.0 | 3 |
| (c) Mesocosm | |  |  |  |  |  |  |  |
| Control (9) | 40 (±0.0) | 8.11 (±0.01) | 17.7 (±0.2) | 54 | 2444.2 (±6.4) | 470.2 (±39.8) | 39.8 | 9 |
| T (9) | 40 (±0.0) | 8.10 (±0.01) | 19.5 (±0.1) | 54 | 2465.2 (±16.8) | 517.9 (±43.5) | 43.5 | 9 |
| CO₂ (9) | 40 (±0.0) | 8.02 (±0.01) | 17.7 (±0.2) | 54 | 2437.8 (±6.8) | 680.0 (±91.9) | 91.9 | 9 |
| T x CO₂ (9) | 40 (±0.0) | 7.98 (±0.01) | 19.5 (±0.1) | 54 | 2445.0 (±4.9) | 734.3 (±64.4) | 64.4 | 9 |

T = elevated temperature treatment, CO₂ = elevated CO₂ treatment, T x CO₂ = combined elevated temperature and elevated CO₂ treatment, TA = total alkalinity.

**Figure S1:** Mean pH in each mesocosm. No significant differences were found between replicate tanks within treatments as tested with a nested ANOVA (p > 0.05) A 2-way ANOVA showed a significantly reduced pH in the CO_2_ treatments (ANOVA; CO_2_ *F*_1,635_ = 105.5, *P*=0.0003). Letters (a, b) above bars denote significant differences.Error bars represent standard error of the mean.

**
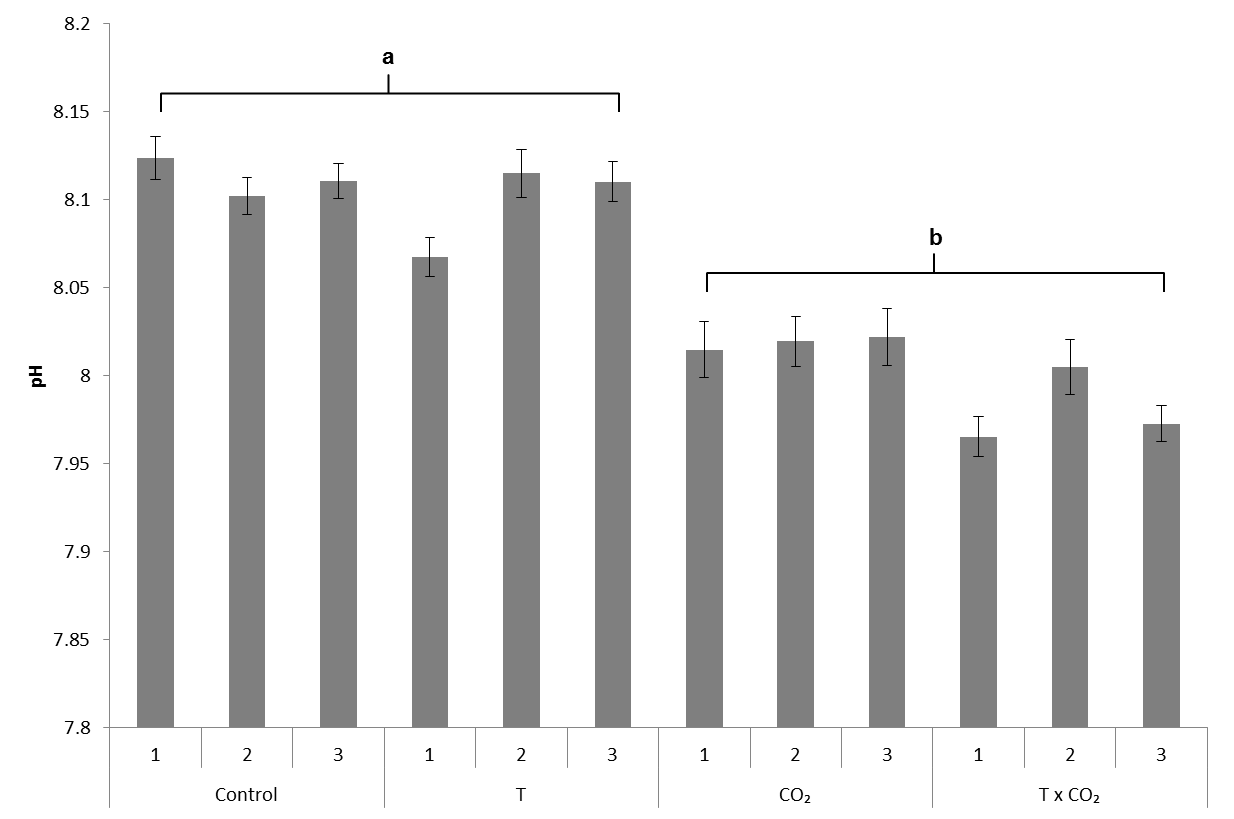
**

**Figure S2:** Diurnal variation of mean pH in each treatment over 3 days in the mesocosm experiment accounting for the variability in pH as seen in Table S2c. Error bars represent standard error of the mean.

**
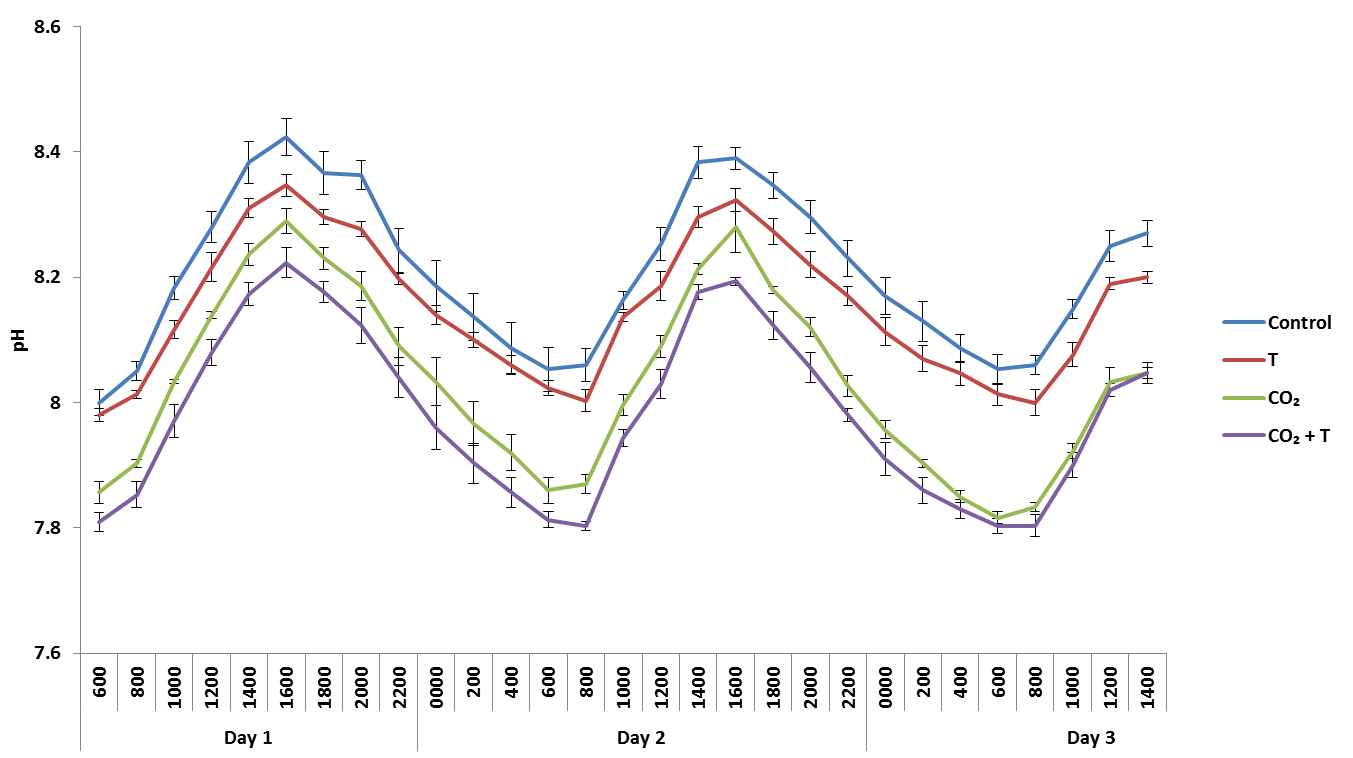
**
